# Editorial: Advances in lung ultrasound: from child to adulthood diseases, volume II

**DOI:** 10.3389/fmed.2025.1650753

**Published:** 2025-08-06

**Authors:** Luigi Vetrugno, Anna Camporesi

**Affiliations:** ^1^Department of Emergency, Anesthesia and Intensive Care, Health Integrated Agency of Friuli Centrale, Tolmezzo, Italy; ^2^Department of Pediatric Anesthesia and Intensive Care, Buzzi Children's Hospital, Milan, Italy

**Keywords:** lung ultrasonography, diaphragm ultrasound, intensive care, pediatrics–children, adult

## Introduction

In the evolving field of respiratory medicine, the diaphragm—often overlooked despite its crucial role in breathing—is finally gaining the recognition it deserves in both scientific research and clinical practice. As our understanding of lung-related diseases deepens, we become better equipped to identify and analyze dysfunctions in diaphragm performance. This advancement is being driven by two significant developments: improvements in ultrasound imaging techniques and the integration of artificial intelligence into clinical workflows. The studies featured in this edition of Frontiers in Medicine highlight a dual revolution in the field. They focus on topics ranging from neonatal respiratory distress to adult hemidiaphragm paralysis, along with the complex challenges posed by pleural effusions. Together, these studies illustrate how technological advancements are expanding the limits of our ability to detect, predict, and understand issues in pulmonary diagnostics.

## Ultrasound beyond structure: toward functional insight

Traditionally, thoracic ultrasound was limited to static imaging, focusing on confirming fluid presence, assessing pleural integrity, and identifying consolidations. However, recent studies are shifting toward functional ultrasonography, particularly regarding diaphragmatic motion and contractility. At the beginning of the pandemic, Vetrugno et al. investigated the role of diaphragm ultrasound in COVID-19 patients during the weaning phase from mechanical ventilation (1). Their multicenter study evaluated the diaphragmatic thickening fraction (DTF) as a potential predictor of weaning outcomes. Although DTF did not prove to be an independent predictor of weaning success or failure, the study highlighted its complex relationship with respiratory effort, sedation levels, gas exchange indices, and the length of ICU stay. Notably, a U-shaped relationship was observed between DTF and oxygenation parameters (PaO_2_/FiO_2_), suggesting that both excessively low and high DTF values could indicate suboptimal respiratory mechanics. These findings emphasize the need for cautious interpretation of DTF values in critically ill patients and reinforce the idea that diaphragm ultrasound should be considered as part of a broader physiological picture. In this edition of Frontiers, Boussuges A. et al. present two significant research studies aimed at standardizing the assessment of diaphragm excursion and inspiratory thickening across various pathological contexts, including hemidiaphragm paralysis and pleural effusion (Boussuges M. et al.). In the study focused on diaphragm paralysis, the research team conducted an in-depth evaluation of over 100 patients who had previously been diagnosed with unilateral diaphragmatic immobility. Their objective went beyond simple observation; they aimed to create a comprehensive framework. By correlating M-mode motion patterns with thickening fractions below 20% and systematically documenting any paradoxical or delayed movements, the researchers established a functional vocabulary for clinicians. They emphasized the importance of preventing misinterpretations—such as confusing delayed activation with paralysis—by combining excursion data with thickening metrics and taking measurements across multiple phases of respiration (Boussuges A. et al.). In patients with pleural effusion, a research group found that fluid accumulation can either mimic or hide diaphragmatic dysfunction (Boussuges M. et al.). This is especially true when large effusions are present, where paradoxical movements are common; however, these movements do not always indicate genuine neuromuscular failure. When patients were reassessed while lying down, and if thickening of the diaphragm was still observed, their function often returned to normal. These findings highlight an important clinical lesson: evaluating diaphragmatic function must be sensitive to the context. Factors such as posture, inspiratory effort, and compensatory mechanisms on the opposite side all influence how we interpret the results. A significant contribution to this evolving field comes from Zhao et al., who established normative reference values for a wide range of multimodal diaphragmatic ultrasound parameters in healthy adults. In addition to traditional measures such as diaphragm thickness and displacement, their study incorporated advanced techniques such as Tissue Doppler Imaging (TDI) and shear wave elastography (SWE), providing a comprehensive functional profile of respiratory muscles. Importantly, the study showed high reproducibility in measurements among different observers, including ultrasound specialists and critical care clinicians, highlighting the potential to standardize these techniques across various clinical environments. These normative data serve as crucial benchmarks for interpreting changes in diaphragmatic function in pathological conditions, further emphasizing the shift toward functional assessment in pulmonary imaging.

## Machine learning in neonatal care: from evaluation to prediction

Ultrasound evaluation provides a real-time view of diaphragm function, while machine learning adds an element of foresight. In their study on neonatal respiratory distress syndrome (NRDS), Huang et al. explore this innovative approach by integrating clinical indicators, such as lung ultrasound scores, oxygenation indices, and assessments of multi-organ function. They use these factors in supervised machine learning models aimed at predicting the severity of the disease. The implications are significant. First, the models provide real-time triage support in neonatal intensive care units (NICUs), where early intervention can be the critical factor between life and death. Second, the algorithms—particularly the random forest model with an AUC of 0.894—outperform traditional logistic regression and even neural networks in terms of accuracy and sensitivity. Moreover, the most predictive variables align with clinical intuition: LUS, OI, RI, and SOFA scores. Here, the strength of machine learning lies not in replacing medical judgment, but in enhancing and accelerating it through statistically validated tools. This research highlights a key point: the effectiveness of AI is heavily dependent on the quality of its inputs. Factors such as the reliability of ultrasound scoring, the accuracy of physiological metrics, and the consistency of measurement protocols all contribute to the success of predictive modeling. Therefore, earlier efforts to refine ultrasound criteria and motion assessments are essential for establishing a solid foundation for these computational approaches.

## Toward a unified diagnostic approach

These four studies collectively present a compelling vision for the future of pulmonary diagnostics—one that is multidimensional, dynamic, and deeply integrated.

1. From descriptive to quantitative:

The transition from subjective image interpretation to objective quantification (e.g., diaphragm thickening fraction, excursion distance in cm, or ultrasound scoring systems) reflects a broader shift in medicine toward evidence-based metrics. These quantifications enable reproducibility, remote consultation, and machine integration—essential components in the era of telemedicine and AI.

2. From static to dynamic assessments:

By evaluating motion, latency, and effort-responsive variables, ultrasound evolves into a tool not only for visualization but also for providing physiological insights. This is particularly evident in the use of anatomical M-mode ultrasonography, which can identify subtle asynchronies and paradoxical patterns that traditional imaging might miss.

3. From diagnostics to prognostics:

The predictive capabilities of machine learning in Neonatal Respiratory Distress Syndrome (NRDS) introduce a new dimension to clinical reasoning—shifting the approach from reactive treatment to anticipatory intervention. When combined with real-time physiological data, these algorithms could change not only how we treat conditions but also when and why we initiate treatment.

4. From organ-based to system-based thinking:

These studies recognize that diaphragmatic dysfunction is rarely an isolated issue. Whether caused by trauma, surgery, sepsis, or congenital immaturity, it affects and reflects systemic physiology. The integration of multi-organ assessment tools, such as the SOFA score in neonatal models, exemplifies this systems approach.

## Challenges and future directions

While the potential of these innovations is promising, they encounter several significant challenges that must be addressed. One major hurdle is the extensive training required for the precise acquisition and interpretation of diaphragm ultrasonography. Mastering this technique requires specialized expertise that is not easily attained. Additionally, variability in assessments among different operators can create inconsistencies, which undermines the reliability of both clinical evaluations and the data used in machine learning models. To overcome these challenges, the development of standardized training programs, comprehensive certification protocols, and innovative automated measurement tools will be crucial in ensuring consistent and accurate results across the board. Such advancements will bridge the gap in expertise and enhance the efficacy of these promising technologies. One of the challenges is accessibility. While major medical centers may have advanced ultrasound systems and AI integration, many smaller or resource-limited hospitals do not. It is crucial to develop simplified, portable, and affordable tools with built-in analytics to ensure equitable implementation worldwide. The ethical landscape of machine learning in medicine requires careful consideration. It is essential to address concerns related to data privacy, algorithmic bias, and clinical responsibility in a transparent and proactive manner. As AI increasingly plays a role in decision-making, it is crucial to maintain a human-in-the-loop approach, ensuring that clinicians remain accountable for their decisions. This approach is not only wise but also necessary.

## Conclusion: the beginning of functional respiratory imaging

In the era of precision medicine, the diaphragm—this seemingly simple sheet of muscle—emerges as a complex diagnostic frontier. Whether affected by surgery, fluid overload, infection, or immaturity, dysfunction of the diaphragm reveals not just issues with breathing but also insights into systemic resilience and vulnerability. Ultrasound, used functionally, provides a valuable lens through which we can understand these conditions. Moreover, artificial intelligence helps to contextualize, project, and personalize our findings. These studies do more than enhance our understanding; they reshape our diagnostic approach. They remind us that innovation in medicine often arises not from discovering new organs, but from reinventing how we perceive and interpret the ones we have always known.

## References

[1] Vetrugno L Orso D Corradi F Zani G Spadaro S Meroi F. Diaphragm ultrasound evaluation during weaning from mechanical ventilation in COVID-19 patients: a pragmatic, cross-section, multicenter study. Respir Res. (2022) 23:210. 10.1186/s12931-022-02138-y 35989352 PMC9392990

